# IgG4-related cholecystitis misinterpreted as gallbladder cancer, a case report

**DOI:** 10.1016/j.amsu.2022.103615

**Published:** 2022-04-10

**Authors:** Seyed Amir Miratashi Yazdi, Elham Nazar, Behnoud Vesali

**Affiliations:** a Department of General Surgery, Sina Hospital, Tehran University of Medical Sciences, Tehran, Iran; b Department of Pathology, Sina Hospital, Tehran University of Medical Sciences, Tehran, Iran

**Keywords:** IgG4-related disease, Gallbladder, Cholecystectomy

## Abstract

**Introduction:**

Immunoglobulin G4-related disease (IgG4-RD) is a new defined entity with features that factually have been overlapped with other diseases with distinctly diverse treatments and prognoses.

**Case presentation:**

This report describes a 50-year-old woman who presented with abdominal pain from 3 months ago. The patient underwent open cholecystectomy with Roux-en-Y hepaticojejunostomy due to suspicious of malignancy. The histological examinations revealed acute on chronic cholecystitis with extensive fibrosis and many inflammatory cells infiltration composed of plasmacells and eosinophils without any evidence of malignancy. Pathological and immunohistochemical examination for IgG4 compatible with IgG4-RD. So, pathological assessment is essential for the diagnosis.

**Conclusion:**

IgG4-RD in gastrointestinal tract frequently misinterpret as malignancy before surgery, and surgeon should notice this disease in the differential diagnosis in order to choose the treatment.

## Highlights

• IgG4-related disease is rare fibroinflammatory condition.
• Gastrointestinal IgG4-related disease is problematic to establish previous surgery.
• Malignancy is main differential diagnosis of IgG4-related cholecystitis.

## 1 Introduction

Agha RA, Franchi T, Sohrabi C, Mathew G, for the SCARE Group. The SCARE 2020 Guideline: Updating Consensus Surgical CAse REport (SCARE) Guidelines, International Journal of Surgery 2020; 84:226–230.

Immunoglobulin type gamma 4-related disease (IgG4-RD) is a systemic disease considered as chronic inflammation with fibrosis and IgG4-positive plasma cells infiltration which responds acceptable to steroids [1]. These disease was first informed in patients with autoimmune pancreatitis in 2001 [2]. Its frequency in the overall or between different geographic or racial groups is unknown and arises generally in middle aged people. IgG4-RD involves males more than females. The most common symptoms are salivary gland including parotid and lacrimal gland bulging, adenopathy, and autoimmune pancreatitis. Sclerosing cholangitis and retroperitoneal fibrosis are other symptoms with high frequency [3]. IgG4-RD is a discrete and clinically separated disease from other autoimmune disease and has strong consideration by means of a new clinical entity, many questions and difficulties still keep on to be clarified, including its pathogenesis, definite diagnostic criteria, and the importance of IgG4 [4]. Herein we report a patient diagnosed with isolated IgG4 related cholecystitis who presented with right upper quadrant abdominal pain and gallbladder stones.

## 2 Case presentation

**Fig. 1:**
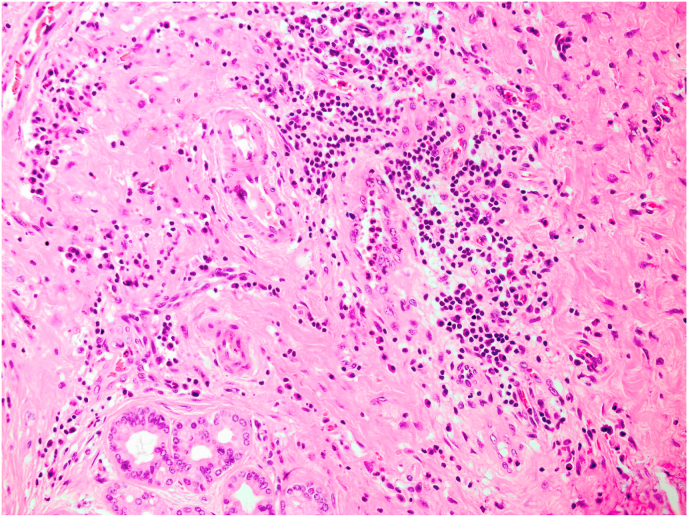
Histopathologic examination showed gallbladder tissue with extensive fibrosis and many inflammatory cells infiltration composed of plasmacells and eosinophils (H&E, x200).

**Fig. 2:**
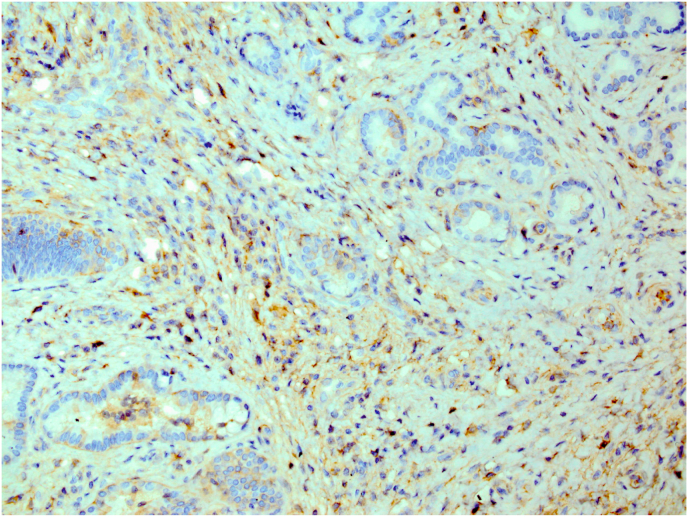
Immunohistochemical staining of IgG4.

A 50-year-old woman was admitted to the emergency department with right upper quadrant abdominal pain lasting 3 months. Family and past medical histories were unremarkable. Routine laboratory investigations were normal. At first, on examination the patient was diagnosed with localized herpes zoster (shingles) on thoracic dermatomes who underwent medical antiviral treatment. After that, the patient had still abdominal patient in second admission and referred to department of surgery. The patient was investigated for reason of pain. Radiologic study shows multiple gallstones on sonography. The patient underwent abdominopelvic computed tomography (CT) scan which revealed increased thickness of gall bladder wall. The patient underwent laparoscopic cholecystectomy. On operation gallbladder noted to be too inflamed and firm in consistency and due to suspicion for malignancy, the laparoscopic procedure plan was changed to open cholecystectomy with Roux-en-Y hepaticojejunostomy. Diagnosis required histopathologic examination. Received specimen for pathology evaluation consists of gallbladder measuring 8cm in length and 2.5cm in diameter. Gallbladder neck diameter was 0.5cm and maximum wall thickness was 0.7cm. On cut section multiple yellowish stones M:2cm in greatest diameter was seen. The pathology evaluation discovered that acute on chronic cholecystitis with extensive fibrosis and many inflammatory cells infiltration composed of plasmacells and eosinophils without any evidence of malignancy (Fig. 1). These findings may be due to IgG4-RD. So, immunohistochemistry (IHC) study for IgG4 was done and showed positive in plasmacells (Fig. 2). After surgery, the patient was discharged in good general condition. The patient received no additional therapy after cholecystectomy. After 6 months from surgery, the patient is still asymptomatic in follow-up examinations. This case report has been reported in line with the SCARE Criteria [5].

## 3 Discussion

IgG4-RD is a newly known fibro-inflammatory disorder with lymphoplasmacytic infiltration and fibrosis of various organs on histological examination [6]. The disease range is very different and is from limited disease with asymptomatic lesion to expanded multi-systemic forms that necessitate quick immunosuppressive treatment [7]. Histopathology examination need to discriminate IgG4-RD from other inflammatory conditions or malignancy. Also, IgG4-RD might has obliterative phlebitis and as differential diagnosis, vasculitis should be mentioned [8]. Gastrointestinal IgG4-RD seems to be problematic to establish previous to surgery because of its uncommonness, and the parallel of its features to neoplastic conditions [1]. Few studies reported the patients who presenting by symptoms of typical cholecystitis, including fever or right upper quadrant abdominal pain. Also, imaging features usually described malignancy as main differential diagnosis. IgG4-related cholecystitis usually accompanies by IgG4-related pancreatitis and sclerosing cholangitis; cases of cholecystitis without other organ involvement are infrequent [9]. So, our case is rare condition. The case reported here was a patient of IgG4-related cholecystitis alone; thus, it should be categorized as a rare case. Inoue et al., reported a case of localized IgG4-cholecystitis misinterpret as gallbladder carcinoma and can't rule out possibility of gallbladder cancer thus performed radical cholecystectomy. Although limited IgG4-related cholecystitis is really rare, discriminating this condition from gallbladder carcinoma is very problematic [10]. This case was similar to our patient which on surgery, laparoscopic cholecystectomy changed to open cholecystectomy due to suspicious for malignancy. Glucocorticoids characterized the first-line choice for induction of remission. Recurrence happens after steroid tapering [11]. IgG4-RD is a fibro-inflammatory disease with an indistinct pathophysiological mechanism which effects on different organs. IgG4-RD can be reason of fibrosis and permanent organ impairment if untreated [12]. IgG4-RD diagnosis is still a clinical challenge and no diagnostic criteria approved. Consequently, any lesion in cases of IgG4-RD, a malignant process is often suspected on early presentation. Surgeons should mention IgG4-RD in the differential diagnosis to avoid needless surgery [13]. Histopathological and IHC examination on biopsy specimens from suspected mass and usage of several imaging modalities might support us to diagnose the disease without surgical resection, and treated by steroid administration. But, surgical resection for IgG4-RD might still be obligatory for patients with worries about neoplastic process. It is problematic to discriminate IgG4-related cholecystitis from gallbladder carcinoma in cases only have localized increased gallbladder wall thickness. In similar cases, surgery with malignancy in mind might be done based on existing clinical data.

## 4 Conclusion

IgG4-RD in gastrointestinal tract frequently misinterpret as malignancy before surgery, and most of the cases were not identified pre-operatively, and surgery was consequently done. So, surgeon should notice this disease in the differential diagnosis to consider appropriate treatment strategies, including surgical resection.

## Ethics approval

Not applicable.

## Consent

Written informed consent was obtained from the patient for publication of this case report and accompanying images. A copy of the written consent is available for review by the Editor-in-Chief of this journal on request.

## Authors' contributions

**SMY:** surgeon performing the operation, major contribution of the idea, study design and revise the paper**, EN:** pathologist and writing the paper**, BV:** data collection and follow up.

## Provenance and peer review

Not commissioned, externally peer-reviewed.

## Declaration of competing interest

There is no conflict to be declared.

## Sources of funding

No source to be stated.

## Registration of research studies

1. Name of the registry:
2. Unique Identifying number or registration ID:
3. Hyperlink to your specific registration (must be publicly accessible and will be checked):

## Guarantor

Elham Nazar is Guarantor of this submission.

## Declaration of competing interest

None.
